# A Maximum-Information-Minimum-Redundancy-Based Feature Fusion Framework for Ship Classification in Moderate-Resolution SAR Image

**DOI:** 10.3390/s21020519

**Published:** 2021-01-13

**Authors:** Gaoyu Zhou, Gong Zhang, Biao Xue

**Affiliations:** Key Laboratory of Radar Imaging and Microwave Photonics, Nanjing University of Aeronautics and Astronautics, Nanjing 211106, China; zhou_gaoyu@nuaa.edu.cn (G.Z.); xuebiao@nuaa.edu.cn (B.X.)

**Keywords:** moderate-resolution SAR image, feature fusion, filter method, kernel principal component analysis (KPCA), maximum-information-minimum-redundancy (MIMR), ship classification

## Abstract

High-resolution synthetic aperture radar (SAR) images are mostly used in the current field of ship classification, but in practical applications, moderate-resolution SAR images that can offer wider swath are more suitable for maritime surveillance. The ship targets in moderate-resolution SAR images occupy only a few pixels, and some of them show the shape of bright spots, which brings great difficulty for ship classification. To fully explore the deep-level feature representations of moderate-resolution SAR images and avoid the “dimension disaster”, we innovatively proposed a feature fusion framework based on the classification ability of individual features and the efficiency of overall information representation, called maximum-information-minimum-redundancy (MIMR). First, we applied the Filter method and Kernel Principal Component Analysis (KPCA) method to form two feature subsets representing the best classification ability and the highest information representation efficiency in linear space and nonlinear space. Second, the MIMR feature fusion method is adopted to assign different weights to feature vectors with different physical properties and discriminability. Comprehensive experiments on the open dataset OpenSARShip show that compared with traditional and emerging deep learning methods, the proposed method can effectively fuse non-redundant complementary feature subsets to improve the performance of ship classification in moderate-resolution SAR images.

## 1. Introduction

Synthetic aperture radar (SAR) is widely used in maritime surveillance and ship monitoring due to its all-time and all-weather observing ability that covers wide areas [1]. Knowledge about the location and type of ship targets can be applicable to multiple applications, such as maritime traffic management, oil spill pollution detection, and illegal smuggling monitoring [2]. Ship classification in SAR images has been studied in depth and has developed into an important part of many operable marine monitoring systems.

Since the swath of SAR images is inversely proportional to image resolution, high-resolution SAR images are usually obtained at the expense of narrow swath. A high-resolution SAR image at the resolution of about 1 m usually corresponds to a swath of 5 km. Such narrow swath may be suitable to inspect specific locations on Earth, but are unsuitable for maritime surveillance. The current satellite-borne SAR can provide swaths of 100–450 km at image resolution of 10–30 m [3], which is more suitable for maritime surveillance in actual situations. In this context, we propose an effective method for ship classification in moderate-resolution SAR images.

Unlike optical images, SAR images are essentially the spatial distribution of electromagnetic wave reflection intensity. The challenges and difficulties of ship target recognition in satellite-borne SAR images are summarized as follows [4]: first, the imaging scene is large, resulting in densely distributed, numerous types of sea targets; second, the imaging distance is long, resulting in extremely low echo signal-to-noise ratio, so the ship targets are not obvious; third, the imaging environment is complex, which brings about a problem that the imaging quality is significantly reduced under high sea conditions, especially moving targets. The most intuitive result of the above problems is that the ship target occupies only a few pixels in a moderate-resolution SAR image and the visual features are extremely inconspicuous, which brings great difficulties to the maritime target recognition [5,6]. Therefore, how to extract the robust features of the maritime target in the complex sea surface environment is the key to the ship target recognition technology.

The typical SAR image ship target recognition is mainly divided into four stages: pre-processing, target detection, feature extraction, and target classification. Among them, feature extraction is a key factor that affects the ship classification in SAR images, and the selection of features directly determines the accuracy of classification. The widely used features in moderate-resolution SAR images are physical features, such as geometric structure features [7], gray-scale statistical features and so on. Lang et al. [3] proposed naive geometric features (NGFs) for ship classification in moderate-resolution SAR images, which are all derived from two basic features: length and width. It’s discovered that when the feature dimension exceeds a certain value, the performance of the classifier will gradually deteriorate, which is called the “curse of dimensionality” [8] in the field of machine learning. M. Dash et al. [9] proposed that a typical feature selection method has four basic steps: generation procedure, evaluation function, stopping criterion, and validation procedure. Kira et al. [10] defined that under ideal conditions, feature selection is to find the minimum feature subset that is necessary and sufficient to identify the target. The existing feature selection methods can be mainly divided into Filter methods and Wrapper methods [11], which have played an important role in the feature selection stage of target classification in SAR images, and have been active for a long time in the field of ship classification [12,13,14,15]. The Filter methods evaluate the predictive ability of each feature according to a certain criterion, thereby selecting several “better” features to form a feature subset. Its significant advantage is that it can quickly remove some non-critical noise features and can be used as a feature pre-selector. The Wrapper methods train the subsequent classifiers with the selected feature subset directly, and evaluate the feature subset according to the classification performance of the test set. It relies on subsequent classifiers, and iteratively trains the classifiers to make it computationally expensive, and the computational efficiency is lower than that of the Filter method.

These algorithms are intuitive and efficient, and they do not change the essential attributes of features, but rely on the accuracy and stability of feature evaluation criteria. Therefore, more nonlinear dimensionality reduction methods have emerged, such as the kernel principal component analysis (KPCA) method [16]. KPCA is one approach of generalizing linear Principal Component Analysis (PCA) into a nonlinear case using the kernel method and it has shown a significant performance in SAR image classification [17,18]. KPCA utilizes the potential nonlinear characteristics of the data in the feature space by introducing an appropriate kernel function in the vector inner product calculation process, and the specific representation of nonlinear mapping does not need to be known, which greatly reduces the amount and complexity of calculation.

With the rapid development of artificial intelligence technology, numerous deep learning (DL) methods have been developed for SAR target recognition. The most commonly used is convolutional neural network (CNN), but it heavily relies on the quality and quantity of training data [19], which is not suitable for our research topic of moderate-resolution SAR image classification in this article. In [19], CNN and metric learning are combined to perform SAR image classification tasks and get a recognition accuracy of 83.67% on the OpenSARShip dataset. Shao et al. [20] proposed a channel-wise and spatial attention (CSA) block which introduces channel attention and spatial attention mechanisms at the same time to enhance the feature extraction ability, and achieved an accuracy of 84% on the OpenSARShip dataset. Recently, a DL algorithm that does not have high requirements for the amount of training data has emerged, that is, the Stacked Autoencoder (SAE). It is a stack of the shallow autoencoder (AE) model, and each layer is based on the expression features of the previous layer. It is an unsupervised learning framework that uses a backpropagation algorithm to reconstruct the input at the output while minimizing the reconstruction error, and performs better than AE. Chen et al. [21] proposed a deep feature extraction method based on SAE and perform classification on hyperspectral data sets, achieving an average accuracy of 94%. Gadhiya et al. [22] applied a SAE to reduce the dimensionality of input feature vector while retaining useful features of the input for multi-frequency PolSAR image. The lowest classification accuracy can reach 91% when using the softmax classifier, which also proves the effectiveness and superiority of SAE in SAR image classification. In this article, the SAE network will be used for comparative experiments.

Based on the above analysis, we consider the feature extraction problem from two perspectives, one is to extract the most separable features, and the other is to extract the most robust and effective features. Therefore, the Filter method is applied to evaluate features and the features with better classification capabilities are selected into the feature subset. And the KPCA method is applied to retain the principal components of the original features and maximize the information representation efficiency of the feature subset to the target. Then, the maximum-information-minimum-redundancy (MIMR) method is utilized to perform reliable feature fusion to maximize information representation and minimize feature redundancy, and different classifiers are selected for ship classification. In Section 2, we will introduce the formation process of two different feature subsets after dimensionality reduction, including the Filter method and KPCA method. The feature fusion framework based on the MIMR method is presented in Section 3. In Section 4, we will first introduce our experimental dataset, and we will present the results of experiments. And Section 5 concludes our work and puts forward future research directions.

## 2. Materials and Methods

**Figure 1:**
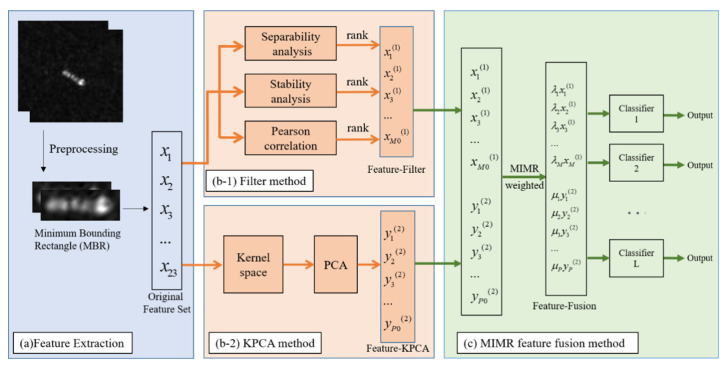
Overall block diagram based on the maximum-information-minimum-redundancy (MIMR) multi-feature fusion framework.

The overview of the basic framework is shown in Figure 1. It mainly includes three parts: feature extraction, feature dimensionality reduction, and feature fusion. For the feature extraction part, taking into account the characteristics of the experimental data in this article, that is the lower resolution results in the inconspicuous structural details of the ship, and faces the problem of imbalance in the data set among various categories, we extract the following features to form the original feature set $F_{0}=\left\{x_{1},x_{2},x_{3},\ldots ,x_{23}\right\}$ after preprocessing the SAR images and extracting the region of interest (ROI).

- Geometric structure features [23]: area $x_{1}$, perimeter $x_{2}$, shape complexity $x_{3}$, length $x_{4}$, width $x_{5}$, aspect ratio $x_{6}$, the maximum distance from the center of mass to the target pixel $x_{7}$, and the average distance from the center of mass to the target pixel $x_{8}$.
- Brightness features [23]: quality $x_{9}$, mean $x_{10}$, and standard deviation $x_{11}$.
- Texture features [23]: energy $x_{12}$, entropy $x_{13}$, moment of inertia $x_{14}$, correlation $x_{15}$, and moment of inverse difference $x_{16}$.
- Moment invariant features [24]: 7 Hu moment invariant features with translation, rotation and scale invariance $x_{17}∼x_{23}$.

Generally, the original feature value has a large range. For example, $x_{8}$ may be as high as $10^{4}$, while the variation range of $x_{12}$ is (0,1). In order to eliminate the negative impact of the feature range on the classification effect, we first normalize the extracted features. Then, we adopted two feature dimensionality reduction methods to obtain two feature subsets in linear space and non-linear space, $F_{Filter}$ and $F_{KPCA}$, to reduce the redundancy between features and ensure the maximum efficiency of the information representation of the target.

### 2.1. Filter Method

In the Filter method, evaluation criteria based on distance measurement, information measurement, and dependency measurement are applied to feature evaluation. In this article, we evaluate original features by three evaluation criteria and the comprehensive ranking method is utilized to select several better features as a candidate feature subset.

- Separability

The separability metric uses the distance between samples to measure the separability of features for different types of samples. The most commonly used distance measures are intra-class distance and inter-class distance. The definition of these two distance measures is expressed as [10]

(1) $$S_{W_{i}}=\sum_{\omega =1}^{3}\frac{N_{\omega }}{N}\left(\frac{1}{N_{\omega }}\sum_{s=1}^{N_{\omega }}{\left(x_{i}-E\left(x_{i}\right)\right)}^{2}\right)$$

(2) $$S_{B_{i}}=\sum_{\omega =1}^{3}\frac{N_{\omega }}{N}\left({\left(E\left(x_{i}\right)-E_{x_{i}}\right)}^{2}\right)$$

where i is the feature label and ω is the type of ships. $E_{x_{i}}=\frac{1}{N}\sum_{\omega =1}^{3}\sum_{s=1}^{N_{\omega }}x_{i}$ is the mean vector of all the samples; $N_{\omega }$ is the number of the $\omega_{th}$ type samples and N is the number of all the samples. The intra-class distance and inter-class distance measures reflect the structural information spread by the sample from different levels, and the most commonly used digital feature for feature selection is the ratio of the intra-class distance to the inter-class distance, which is defined as [25]:

(3) $$J_{i}=\frac{S_{B_{i}}}{S_{W_{i}}}$$

We hope that features can effectively distinguish different target categories, that is, the smaller the intra-class distance is, the better, and the larger the inter-class distance is, the better. Therefore, the larger $J_{i}$ of the feature is, the better its separability is.

- Stability

A subset of features for which a classifier obtains the best results may not necessarily be applicable to other classifiers. Therefore, the stability of the feature itself is also a factor that must be paid attention to in feature selection. The normalized coefficient of variance is used to measure the stability of the target feature, which is defined as:

(4) $$ST_{i}=\frac{E\left(x_{i}{}^{2}\right)-E^{2}\left(x_{i}\right)}{E\left(x_{i}{}^{2}\right)}$$

where $E\left(x_{i}{}^{2}\right)$ is the mean square of the feature and $E^{2}\left(x_{i}\right)$ is the square of the mean of the feature. The smaller the normalized variance coefficient of the feature $ST_{i}$ is, the more stable the feature is [25].

- Pearson correlation coefficient

In statistics, the Pearson correlation coefficient is often used to measure the degree of linear correlation of variables, which is defined as the ratio of covariance to standard deviation. The greater the absolute value of the correlation coefficient, the stronger the correlation, that is, the closer the correlation coefficient is to 1 or −1, the stronger the correlation; the closer the correlation coefficient is to 0, the weaker the correlation, which is defined as:

(5) $$\rho_{i,j}=\frac{\sum x_{i}x_{j}-\frac{\sum x_{i}\sum x_{j}}{N}}{\sqrt{\left(\sum x_{i}{}^{2}-\frac{{\left(\sum x_{i}\right)}^{2}}{N}\right)\left(\sum x_{j}{}^{2}-\frac{{\left(\sum x_{j}\right)}^{2}}{N}\right)}}$$

Obviously, the lower the $\rho_{i,j}$ is, the smaller the redundancy between features $x_{i}$ and $x_{j}$ is, and the more they should be selected into the candidate feature subset.

After feature extraction, we first analyze the separability, stability and Pearson correlation coefficient of features. Then we comprehensively evaluate the features according to their separability and stability, and subtract the features when the separability is lower than a certain threshold and the stability is higher than a certain threshold. Next we analyze the correlation coefficients of the remaining features, and select the features with lower correlation with other features into the feature subset. Finally, a feature vector is constructed for each ship chip: $F_{Filter}=\left\{x_{1}{}^{\left(1\right)},x_{2}{}^{\left(1\right)},x_{3}{}^{\left(1\right)},\ldots ,x_{M0}{}^{\left(1\right)}\right\}$.

### 2.2. KPCA Method

An initial motivation of KPCA is to perform PCA in the high-dimensional feature space. As we know, PCA minimizes the distance between the data and the linear principal component; while KPCA first selects a suitable nonlinear mapping, and then maps the original data into a high-dimensional nonlinear feature space F, to minimizes the distance between the data and the nonlinear principal component [16]. KPCA constructs an optimal plane in this high-dimensional space to make the mapped data separable. Then PCA is performed on the data mapped to the high-dimensional space to obtain a better classification and recognition effect.

Given the sample set $X=\left\{x_{1},x_{2},\ldots ,x_{i},\ldots ,x_{L}\right\}\;\left(i=1,2,\ldots ,L\right)$, where $x_{i}\in R^{n}$ is a vector of n dimension, $R^{n}$ is the put data space and L is the total number of samples. The pre-selected nonlinear mapping is Φ, and the high-dimensional nonlinear mapping space after mapping is F, so the samples in the feature space is donated by:

(6) $$\Phi \left(X\right)=\left\{\Phi \left(x_{1}\right),\Phi \left(x_{2}\right),\ldots ,\Phi \left(x_{L}\right)\right\}$$

Kernel techniques are introduced to avoid huge calculations. K is a L×L kernel matrix which is defined by the inner product:

(7) $$K_{ij}=K\left(x_{i},x_{j}\right)=\Phi^{T}\left(x_{i}\right)\Phi \left(x_{j}\right)$$

The covariance matrix on the feature space F can be constructed by:

(8) $$C=\frac{1}{L}\sum_{i=1}^{L}\Phi \left(x_{i}\right)\Phi {\left(x_{i}\right)}^{T}$$

The corresponding eigenvalue equation is:

(9) Cv=λv

The principal components are decided by the eigenvalue λ and the eigenvector v of the covariance matrix C. According to the reproductive kernel theorem, that is, any vector in the space (even the base vector) can be linearly represented by all samples in the space. So the eigenvector v can be expressed as:

(10) $$v=\sum_{i=1}^{L}\alpha_{i}\Phi \left(x_{i}\right)$$

Therefore, Equation (9) can be transformed to:

(11) $$K\alpha =\lambda^{\prime }\alpha$$

where $\lambda^{\prime }=L\lambda$ are the eigenvalues. The eigenvalues are obtained and sorted in descending order $\lambda_{1}{}^{\prime }\geq \lambda_{2}{}^{\prime }\geq \ldots \geq \lambda_{n}{}^{\prime }\;$, and the corresponding eigenvectors are $\alpha_{1},\alpha_{2},\ldots ,\alpha_{n}$. We select the first P0 principal components $\alpha_{1},\alpha_{2},\ldots ,\alpha_{P0}$ according to the cumulative contribution rate of the eigenvalues. Then we calculate the projection Y=Kα of the sample X, and Y is the original data after dimensionality reduction, that is, the reduced feature vector $F_{KPCA}=\left\{y_{1}{}^{\left(2\right)},y_{2}{}^{\left(2\right)},y_{3}{}^{\left(2\right)},\ldots ,y_{P0}{}^{\left(2\right)}\right\}$ for each ship-chip.

The kernel function provides a connection from linear to nonlinear and any algorithm that can represent the dot product between two vectors. The specific representation of mapping Φ does not need to be known, so the amount of calculation is greatly reduced, and the complexity is also eased. Here we choose the Gaussian kernel function with better learning ability.

## 3. MIMR Feature Fusion Method

In addition to using a certain feature alone, the rational use of different types of features combined with each other can achieve complementary effects. The most important problem to be solved is how to effectively combine different types of features, which is called feature-level fusion [26]. Feature-level fusion can not only increase the feature information of the image, but also effectively integrate the advantages between different features and improve the adaptability of the algorithm. At present, the existing feature fusion algorithms are mainly divided into three categories: feature combination, feature selection, and feature conversion [27,28]. A simple feature combination may result in useless redundant information between various dimensions, which will affect the performance of the classifier. Therefore, we need to consider that the number of feature subsets is as low as possible to efficiently represent the target, while taking into account the low redundancy between features. Inspired by the minimal-redundancy-maximal-relevance (mRMR) method [25], this paper proposes a maximum-information-minimum-redundancy (MIMR) feature fusion method.

The idea of the MIMR method is to consider the information representation efficiency of the target feature subsets with different physical attributes while minimizing the redundancy between feature vectors $F_{Filter}$ and $F_{KPCA}$. This coincides with our original intention, which is to reduce the redundancy of different feature vectors while ensuring classification accuracy. Various features are normalized before fusion, which is of great significance to the improvement of the overall classification effect.

Given the feature set $F_{con}=[F_{Filter},F_{KPCA}]$, and the target type ω, the feature selection problem is to find a feature set $S=[\lambda F_{Filter},\mu F_{KPCA}]$ that “optimally” characterizes ω. The optimal characterization condition often means the maximum percentage of correct classification (PCC), which usually requires the feature set S for classification to contain as much information of the target as possible.

Furthermore, it has been recognized that the combinations of individually good features do not necessarily lead to good classification performance. In other words, there may be redundancy between “good” features. Here we utilize mutual information (MI) to represent redundancy, and minimum redundancy represents the smallest correlation between features, that is, the smallest MI between features. Given two random variables X and Y, their MI is defined in terms of their probabilistic density p(x), p(y), and p(x,y):

(12) $$I(X;Y)=\sum_{x\in X}\sum_{y\in Y}p(x,y)\log\frac{p(x,y)}{p(x)p(y)}$$

Therefore, the optimization model can be established as

(13) $$\begin{array}{l} \underset{\lambda_{i},\mu_{j}}{\min}R=\sum_{i=1}^{M}\sum_{j=1}^{P}I(\lambda_{i}x_{i},\mu_{j}y_{j})\\ s.t.\left\{ \begin{array}{l} \sum_{i=1}^{M}\lambda_{i}=1\\ \sum_{j=1}^{P}\mu_{j}=1 \end{array}\right. \end{array}$$

where $\left\{x_{i},i=1,2,\ldots ,M\right\}$ and $\left\{y_{j},j=1,2,\ldots ,P\right\}$ are the feature vectors in $F_{Filter}$ and $F_{KPCA}$.$\left\{\lambda_{i},i=1,2,\ldots ,M\right\}$ and $\left\{\mu_{j},j=1,2,\ldots ,P\right\}$ are the weight coefficients of a single feature vector in $F_{Filter}$ and $F_{KPCA}$.The requirement to solve this optimization model is actually to solve a constrained nonlinear multivariable function [29]. The idea of solving this optimization model is to find a set of optimal feature weight vectors $\left\{\lambda_{1},\lambda_{2},\ldots ,\lambda_{M},\mu_{1},\mu_{2},\ldots ,\mu_{P}\right\}$ through iterative optimization, so that the MI, that is, the redundancy between feature vectors is minimized while ensuring PCC. Finally, an optimal fused feature vector will be constructed by $S=\left\{\lambda_{1}x_{1},\lambda_{2}x_{2},\ldots ,\lambda_{M}x_{M},\mu_{1}y_{1},\mu_{2}y_{2},\ldots ,\mu_{P}y_{P}\right\}$.

To further improve efficiency, we first adopted the Fisher Score method [29] to make a preliminary judgment on the concatenated features $F_{con}$. According to the Fisher criterion, when the Fisher Score of the selected feature is higher, it means that the feature makes the distance between different categories in the sample points farther, the closer the distance between samples of the same category, and the classification ability of the feature is better. The Fisher Score of the $i_{th}$ feature is defined as [30]:

(14) $$FS_{i}=\frac{\sum_{\omega =1}^{3}N_{\omega }{\left(E(x_{i})-E_{x_{i}}\right)}^{2}}{\sum_{\omega =1}^{3}N_{\omega }\sigma_{i}^{2}}$$

where i is the feature label and ω is the type of ships. $E_{x_{i}}$ is the mean vector of all the samples, and $\sigma_{i}^{2}$ represents the variance of the $\omega_{th}$ type sample corresponding to the $i_{th}$ feature variable. When the Fisher Score of a feature is extremely low (lower than the threshold), the feature will be subtracted. Then we perform the MIMR feature fusion method on the remaining features.

In terms of classifier selection, we consider several mature classifiers including: k-Nearest Neighbor (KNN) classifier based on Euclidean distance, support vector machine (SVM) classifier based on kernel function, and neural network classifier based on artificial intelligence [31,32,33]. KNN classifier is simple to calculate and easy to execute, making it the most commonly used classifier in target recognition; SVM classifier is a new machine learning method aiming at finding the optimal classification surface. Due to its excellent learning ability, it has also been widely used in SAR image target classification and recognition tasks; neural network classifier for ship classification in SAR images has become a boom, but the lack of a sufficient number of labeled class-balanced databases limits its application and development.

Considering that the number of samples between the categories of experimental data in this article is extremely unbalanced, it is difficult for neural network classifiers to train a good network model, so here we choose KNN classifier and SVM classifier to ensure the classification accuracy and the speed of the algorithm, and we use LibSVM [34] implementation.

The detailed process to obtain the weight coefficients $\left\{\lambda_{1},\lambda_{2},\ldots ,\lambda_{m},\mu_{1},\mu_{2},\ldots ,\mu_{p}\right\}$ of fused feature vector by the MIMR method is shown in Algorithm 1.

**Algorithm 1** The process of the MIMR algorithmStep 1. After normalizing the feature vectors $F_{Filter}$ and $F_{KPCA}$, perform feature combination to obtain a feature subset $F_{con}=[F_{Filter},F_{KPCA}]$. Step 2. Utilize the Fisher Score method to evaluate the $M_{0}+P_{0}$ features in $F_{con}$,we get $\left\{FS_{1},FS_{2},\ldots ,FS_{K}\right\}$. Sets the threshold ε, when $FS_{k}<\varepsilon$, the feature $x_{k}$ are subtracted. Step 3. For the remaining M+P features, initialize the weight vectors of a single feature vector $\sum_{i=1}^{M}\lambda_{i}{}^{0}=1,\sum_{j=1}^{P}\mu_{j}{}^{0}=1$ (constraints). Step 4. Calculate the mutual information between the M features of $F_{Filter}$ and P features of $F_{KPCA}$,we will get a M×P MI matrix $I(\lambda_{i}x_{i},\mu_{j}y_{j})$. Then calculate the objective function $R=\sum_{i=1}^{M}\sum_{j=1}^{P}I(\lambda_{i}x_{i},\mu_{j}y_{j})$. Step 5. Utilize iterative optimization method to find the local minimum that satisfies the constraints. Compare the objective function R with the default value of the optimality tolerance ξ. When R>ξ, return to step 3; when R<ξ, go to step 6. Step 6. When objective function R is non-decreasing in feasible directions, that is, reaches minimum, and constraints are satisfied to within the default value of the constraint tolerance, the optimization is completed and $\left\{\lambda_{1},\lambda_{2},\ldots ,\lambda_{M},\mu_{1},\mu_{2},\ldots ,\mu_{P}\right\}$ is obtained.

## 4. Experiment

### 4.1. Dataset

**Figure 2:**
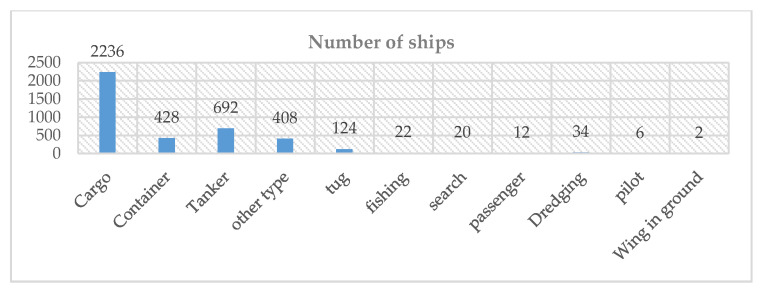
The statistical results of the types of ships in the GRD mode of the dataset.

**Figure 3:**
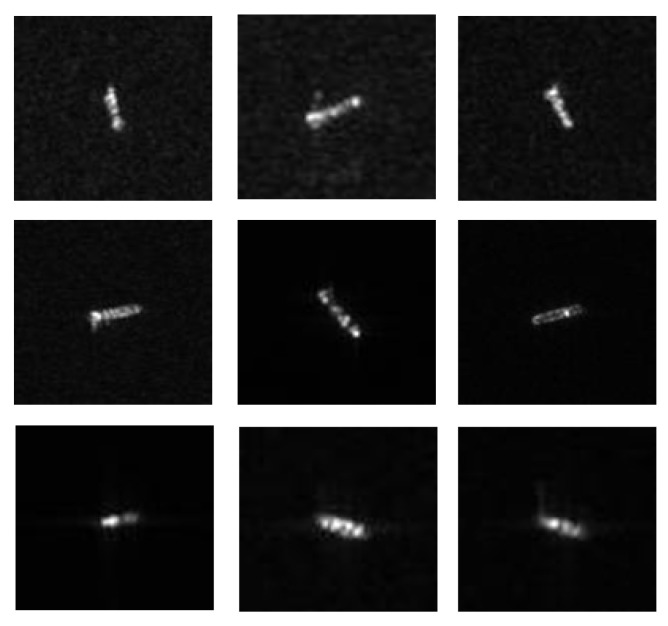
Example of the data set. **Top** to **bottom**: cargo ship; container ship; tanker.

In this article, the experimental dataset selected is the OpenSARShip [35] dataset, which comes from the dual-polarized satellite-borne SAR data detected by the European Space Agency’s Sentinel-1 satellite. It has 11,346 ships and their corresponding Automatic Identification System (AIS) information. The OpenSARShip provides two available products of the interferometric wide swath mode (IW) mode: the single look complex (SLC) and ground range detected (GRD) products, with VV-VH dual polarization. The image spatial resolution in GRD mode is 20 m × 22 m, which fits well with our research topic of moderate-resolution SAR image, so we choose images in GRD mode for the experiments. Figure 2 shows the type distribution of data sets in GRD mode. Figure 3 is an example of the data set. Since many ship chips in the dataset are extremely small in size (smaller than 30 × 30) and are not suitable for classification, we selected three types of ship chips with a suitable size (greater than 70 × 70) for experiment, including 250 Cargo ships, 240 Container ships and 134 Tankers.

### 4.2. Results and Discussion

**Figure 4:**
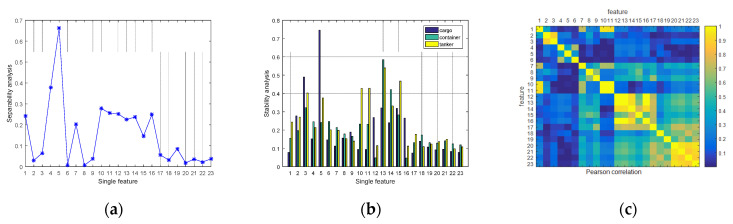
Feature analysis result. (**a**) separability analysis; (**b**) stability analysis; (**c**) Pearson correlation analysis.

After feature extraction and analysis, the separability, stability, and Pearson correlation coefficient analysis result are shown in Figure 4.

From the result, it can be seen intuitively that different features have different properties and distinguishability. For example, the width $x_{5}$ has good separability (as shown in Figure 4a, but it performs poorly in stability on cargo ships (as shown in Figure 4b. In addition, there is a strong correlation between $x_{20},x_{21},x_{22}$ and $x_{23}$ in the Hu moment invariant features (as shown in Figure 4c although they are relatively stable (as shown in Figure 4b, this is the very reason that we need to perform feature selection and fusion. Firstly, $\left\{x_{2},x_{6},x_{8},x_{9},x_{18},x_{20},x_{21},x_{22},x_{23}\right\}$ are subtracted due to their low separability. Secondly, $\left\{x_{3},x_{10},x_{11},x_{13},x_{14},x_{15}\right\}$ are subtracted due to their poor stability. Finally, we evaluate the correlation coefficient between the remaining features and the feature vector after screening is $F_{Filter}=\left\{x_{1},x_{4},x_{5},x_{7},x_{12},x_{16},x_{17},x_{19}\right\}$. Then we combine the feature vector $F_{KPCA}$ obtained by the KPCA method to perform MIMR-based feature fusion for experiments.

In each experiment, we randomly selected 200 container ships, 200 bulk carriers, and 100 tankers as the training set, and the rest as the test set. In order to eliminate the bias caused by random sampling to a single experimental data, the above experiments were carried out 50 times. The performance of the classifier is finally reported as the average classification accuracy. KNN classifier and SVM classifier are selected to perform classification tasks to comprehensively evaluate the method. The recognition result is shown in Table 1.

Where Feature-fusion is the feature vector $S=\left\{\lambda_{1}x_{1},\lambda_{2}x_{2},\ldots ,\lambda_{M}x_{M},\mu_{1}y_{1},\mu_{2}y_{2},\ldots ,\mu_{P}y_{P}\right\}$ obtained by the MIMR method proposed in this article. $F_{G}=\left\{x_{1},x_{2},..,x_{8}\right\}$ and $F_{T}=\left\{x_{9},x_{10},..,x_{16}\right\}$ are the geometric structure features and texture features in the original feature set $F_{0}=\left\{x_{1},x_{2},x_{3},\ldots ,x_{23}\right\}$. $F_{SAE}$ and $F_{mRMR}$ are the feature vectors obtained by the SAE method and the mRMR method respectively, which are regarded as part of the comparative experiments as well. To understand the impact of features with different physical attributes on classification, we first select feature vectors $F_{G}$ and $F_{T}$ to verify the classification ability of the original features we extracted. We can see that $F_{G}$ have a better classification performance than $F_{T}$, this is because texture features reflect the sharpness of the image and the thickness of the texture, which means that it is directly related to the resolution of the image. This also reflects that for the Sentinel-1 products with moderate resolution, the categories of geometric features show better classification performances, which is consistent with our analysis. The feature vector S always has the best performance among all the classifiers, which proves the robustness and stability of our method.

We also found that the same feature vector performs differently on different classifiers. The feature vector $F_{SAE}$ has good performance in Softmax classifier, but it performs poorly in other classifiers. We can conclude that the optimal feature subset may change with the classifier, that is, there may be inherent relevance between the optimal feature subset and the classifier, which can be taken as a future work of our research. After further analyzing the classification results of three different types of ships, we found that the PCC of cargo ships and container ships are higher than that of tankers. This is because the size of tankers varies greatly, which means larger intra-class distance, and tankers account for fewer pixels in ship chips than the other two ship types, which limits the effectiveness of feature extraction and is worthy of our further study.

In addition, we compared the performance of the proposed method with other methods for ship classification in moderate-resolution SAR images, including naive geometric features (NGFs) [3], Geometric features/local radar cross section (LRCS) (three sections) features [34], CNN + Metric [19], Lightweight CNN [20] and Semisupervised learning [36]. The comparison results are shown in Table 2. Compared with the current popular CNN methods, our method does not need to train a complex network framework, nor does it require complex parameter settings. Considering that our experimental data are moderate-resolution SAR images, we pay more attention to geometric structure features and gray-scale statistical features, while discarding those features that require high image resolution. Compared with traditional methods, we choose the Filter method to evaluate features and select “good” features with better classification abilities, and apply the KPCA method to reduce the dimension while retaining the principal components of the original information. Then we propose an MIMR-based feature fusion strategy to minimize the redundancy between features while ensuring the information representation efficiency of the overall feature subset to the target. It can be clearly seen that whether it is the classical machine learning method or the emerging CNN method, the PCC of the proposed method is much higher than them, which proves that our method is simple, feasible, and comparatively robust. Current satellites capable of SAR imaging mainly include TerraSAR-X [2], Sentinel-1 [35], Radarsat-2 [37], and Gaofen-3 [38], which are operating in different imaging modes with different resolution and swath. With comparable resolutions, we believe that the proposed method should be able to perform well in the SAR images obtained from the current satellite missions.

## 5. Conclusions

This article aims to provide a reliable and operable method for ship classification in moderate-resolution SAR images. We extract the motivation of the target classification problem from multi-dimensional feature selection and fusion and correct classification rate. A novel multi-feature fusion framework based on the MIMR method is proposed, which not only considers the classification ability of a single feature, but also considers the information representation efficiency of the overall feature subset to the target, while ensuring that the redundancy is minimized. The results of the comparative experiments show that the proposed method effectively reduces the redundancy between features, and has a fairly good performance for ship targets classification in moderate-resolution SAR images.

The preliminary analysis we presented here can be extended in many ways. We intend to conduct extensive experiments in combination with feature fusion under different polarization channels. In addition, we will continue to study other more advanced classifiers.

## Figures and Tables

**Table 1:** The PCC of different feature vectors in different classifiers.

PCC (%)	KNN Classifier	SVM Classifier
$F_{G}$	87.39	94.35
$F_{T}$	73.29	80.90
$F_{Filter}$	88.24	95.73
$F_{KPCA}$	86.04	90.97
$F_{SAE}$	95.19 (Softmax Classifier)	
$F_{mRMR}$	83.46	92.19
**Feature-fusion (Proposed** **)**	**90.48**	**97.14**

**Table 2:** The PCC of the proposed method and the method for comparison in moderate-resolution SAR images.

Methods	Spatial Resolution of Experimental Data	Ship Type of Classification Result	Classifier	PCC (%)
NGFs [3]	15 m × 15 m	Bulk, containers and tankers	KNN	64.6
			SVM	67.6
			MKL	71.0
Geometric/LRCS (3 sections) [34]	20 m × 22 m	Tanker, container ship, and bulk carrier	Geometric-KNN	76.49
			LRCS (3 sections)-KNN	75.69
CNN + Metric [19]	20 m × 22 m	Tanker, container, and bulk carrier	Softmax	83.67
Lightweight CNN [20]	20 m × 22 m	Cargo, tanker, tug and others	Softmax	84
Semisupervised learning [36]	20 m × 22 m	Bulk carrier, container ship and tanker	Softmax	74.96
**Proposed method**	**20 m × 22 m**	**Cargo, container and tanker**	**KNN**	**90.48**
			**SVM**	**97.14**

## Data Availability

The data presented in this study are openly available in [35].
